# Efficacy of Oral Paracetamol Compared with Oral Ketoprofen for Pain Management in Office Hysteroscopy: A Double-Blind, Randomized Clinical Trial

**DOI:** 10.3390/medicina62010170

**Published:** 2026-01-14

**Authors:** Tricia Dewi Anggraeni, Andika Widyatama, Vivian Soetikno, Gerald Sebastian Davis, Hendra Adibia Setiaka, Maria Christina Sekarlangit

**Affiliations:** 1 Gynecology Oncology Division, Department of Obstetrics and Gynecology, Faculty of Medicine Universitas of Indonesia, Dr. Cipto Mangunkusumo National General Hospital, Jakarta 10430, Indonesia; andika2widyatama@gmail.com (A.W.); geraldsebastiandavis@gmail.com (G.S.D.); hendra.adibia@gmail.com (H.A.S.); mariacsekarlangit@gmail.com (M.C.S.); 2 Department of Pharmacology and Therapeutics, Faculty of Medicine Universitas of Indonesia, Dr. Cipto Mangunkusumo National General Hospital, Jakarta 10430, Indonesia; vivian.soetikno@ui.ac.id

**Keywords:** gynecologic endoscopy, outpatient procedure, visual analogue scale, nonsteroidal anti-inflammatory drugs, women’s health, analgesic efficacy, randomized clinical trial

## Abstract

*Background and Objectives*: Hysteroscopy has become the “gold standard” in assessing uterine cavity abnormalities, and currently it can be performed in an “office setting”. Although office hysteroscopy has a better level of comfort than operative hysteroscopy, pain is a common concern. Nonsteroidal anti-inflammatory drugs (NSAIDs) are frequently used for pre-procedure analgesia, but they may cause gastrointestinal side effects. Paracetamol offers to be a safer alternative, but its efficacy in this setting is limited. This study aimed to compare the efficacy and safety of oral paracetamol with oral ketoprofen for pain management during office hysteroscopy. *Materials and Methods*: Double-blind, parallel-group, randomized controlled trial conducted at a single hysteroscopy center in Jakarta, Indonesia, over a 2-year period. Sixty women undergoing office hysteroscopy were randomized (1:1) to receive paracetamol 1000 mg orally or ketoprofen 100 mg orally 1 h before the procedure. *Results*: All participants completed the trial and were included in the analysis. The median visual analog score (VAS) during the procedure was 2 (range 0–8) in the paracetamol group versus 3 (range 0–6) in the ketoprofen group (*p* = 0.266). Median cramping scores 30 min post-procedure in the paracetamol group were 0 (range 0–5) vs. 0 (range 0–4) in the ketoprofen group, respectively (*p* = 0.499). Side effects occurred in 3 participants (10%) in the ketoprofen group and none of the paracetamol group. Comfort scores were high in both groups (median 9/10). No vagal reflexes were observed. *Conclusions*: Oral 1000 mg paracetamol was as effective as oral 100 mg ketoprofen for pain management during and after office hysteroscopy, with fewer side effects. Paracetamol may be a safe and cost-effective alternative for pre-procedure analgesia in office hysteroscopy.

## 1. Introduction

Hysteroscopy is a procedure for evaluating intrauterine pathological conditions [1]. Hysteroscopy has become the “gold standard” in assessing uterine cavity abnormalities and provides direct diagnostic information with a sensitivity of up to 98% when compared to curettage procedures [1]. Apart from the diagnosis process, hysteroscopy is also useful for the treatment of uterine abnormalities such as abnormal uterine bleeding and subfertility [2].

Currently, hysteroscopy procedures can be performed in outpatient clinics in an “office setting” called office hysteroscopy (OH) [3]. OH can provide many advantages compared to hysteroscopy performed in the operating room (traditional hysteroscopy), including reduced cost of hospital care, the duration of recovery after the procedure, the possibility of complications such as cervical tears or uterine perforation, and not requiring general or regional anesthesia and preoperative preparation so as to increase comfort for the patient [2,4].

Even though it has a better level of comfort than hysteroscopy in the operating room, the OH procedure can still cause pain in the patient [3]. Factors that influence the occurrence of pain in the OH procedure are classified into two, namely factors originating from the patient (patient-related) and from the OH (procedural-related) procedure itself. Factors originating from the patient include parity, menopause, chronic pelvic pain, dysmenorrhea, and history of delivery method (vaginal or cesarean section). Then, factors originating from the procedure include the duration of the procedure, operator experience, and type of equipment [5,6].

Pain is still the main cause of failure in OH procedures, so appropriate analgesics are needed to overcome it [7]. There are several pain management modalities in OH procedures, namely local anesthesia, systemic analgesics, and transcutaneous nerve stimulation (TENS).

According to the Royal College of Obstetricians and Gynaecologists (RCOG), standard doses of nonsteroidal anti-inflammatory drugs (NSAIDs) analgesics can be given approximately 1 h before the OH procedure is carried out to reduce post-procedure pain [8]. However, NSAIDs often cause side effects, most commonly occurring in the digestive tract (reaching 40% of patients given NSAIDs). Symptoms and signs such as nausea, vomiting, dyspepsia, diarrhea and peptic ulcers can be found as side effects in NSAID users [9,10,11]. Study for single doses, patients consistently reported pain relief with NSAIDs with common side effect such as gastrointestinal symptoms, dizziness, and drowsiness [12]. The most common complication is bleeding due to ulcer perforation [13]. According to data from RSUPN Dr. Cipto Mangunkusumo, 30–40% of NSAIDs users were found without complaints even though NSAIDs gastropathy lesions have been found per endoscopy [14]. So far at RSUPN Dr. Cipto Mangunkusumo, NSAID ketoprofen 100 mg was given rectally for pain management for OH procedures. Rectal ketoprofen is given 1–2 h before the OH procedure, causing a longer waiting time for the patient which increases the risk of pain during the OH procedure [15].

It is necessary to consider alternative analgesics with fewer side effects for pain management for OH procedures, as well as more practical drug preparations given to patients. In the RCOG guidelines, it is stated that paracetamol can also be used in OH pain management. However, there is no clinical or research evidence that clearly shows the efficacy of paracetamol in the management of OH pain [16]. This study compared the 1000 mg oral paracetamol group with the 100 mg oral ketoprofen group for pain management of OH procedures. Adequate pain management is expected to increase the success of OH procedures.

## 2. Materials and Methods

This study used a double-blind, randomized clinical trial to assess the intensity of pain during the OH procedure and cramping within 30 min after the OH procedure in the group given paracetamol 1000 mg orally compared with ketoprofen 100 mg orally. This study also evaluated the side effects that occurred in both groups. This research was conducted at the OH clinic at RSUPN Dr. Cipto Mangunkusumo Kintani Jakarta, Indonesia over a period of 2 years. All patients who will undergo an OH procedure are checked for eligibility when they arrive and are offered to be research subjects if they meet the inclusion criteria, namely women who underwent an OH procedure and were not using analgesics in the 1 month before joining the study. Individuals are not eligible if they have exclusion criteria, namely women with a history of asthma and women with a history of allergies to paracetamol or NSAID class drugs. Patients were given an explanation of the research procedures and asked for informed consent, if they were willing to take part in the research. This research was approved by the Health Research Ethics Committee of the Faculty of Medicine, Universitas Indonesia (ND—0054/UN2.F1.DEPT.25/PDP.01/2022) and the Clinical Trial Registration number is NCT07315698.

Research subjects were divided into two groups. The first group received the analgesic paracetamol tablet at a dose of 1000 mg orally and the second group received the analgesic ketoprofen tablet at a dose of 100 mg orally, each consisting of 30 subjects according to the sample size calculation formula. The total sample size of 60 patients (30 per group) was determined based on previously published data and feasibility considerations. According to variability reported in comparable trials by Issat et al. (SD ≈ 1.65) and Terán-Alonso et al. (SD ≈ 2.86), a group size of 30 participants per arm provides approximately 80% power (α = 0.05, two-sided) to detect a mean difference of about 1.2 VAS points under the lower-variance assumption (Issat et al.) or approximately 2.1 VAS points under a more conservative variance (Terán-Alonso et al.) [17,18]. Therefore, a total of 60 participants was considered a reasonable and practical compromise between statistical power and study feasibility. Subject allocation was carried out by block randomization so that an equal number of subjects were obtained in both groups. The block size was determined at 6 subjects. Randomization was carried out by computer and a randomization code was obtained by a research assistant. Then, the medicines are removed from each factory packaging, then put into sterile medicine plastic with serial numbers 1–60 which are kept by the nurse. The nurse handed over the medicine in the plastic according to the serial number to the research subject patient 1 h before the OH procedure. Research subjects and researchers did not know the type of drug given (double blind).

All OH procedures were performed using a Karl Storz Bettocchi Office Hysteroscope (Tuttlingen, Germany) with an outer diameter of 3.5 mm. Vaginoscopy approach was applied, without the use of a speculum or tenaculum. The uterine cavity was distended with 0.9% normal saline at a pressure of approximately 70 mmHg using a continuous flow system. No cervical preparation (such as misoprostol) was administered prior to the procedure. All procedures were conducted by the same experienced operator.

The primary outcome of this study was the intensity of pain during the office hysteroscopy procedure, measured using a VAS at the time when the hysteroscope entered the external cervical ostium. The secondary outcomes included cramping pain within 30 min after the procedure, side effects associated with the administered analgesic, patient comfort, and the occurrence of vagal reflexes.

Assessment of pain intensity during the procedure (when the hysteroscope instrument enters the external cervical ostium) and cramping within 30 min after the OH procedure will use a VAS. Patients are asked to determine the VAS value in the questionnaire according to the intensity of pain and cramping they feel. Side effects of drug administration, comfort, and vagal reflexes will be assessed using the same questionnaire.

Data analysis was carried out using IBM SPSS version 25.0. Data distribution was assessed for normality. The age variable was normally distributed, while the procedure duration, VAS pain, cramping, and comfort scores were non-normally distributed. Between-group comparisons were originally analyzed using unpaired *t*-tests. Although several variables did not follow a normal distribution and non-parametric methods would have been more appropriate, the reported *p*-values remain unchanged, and all results were non-significant. Categorical data (such as side effects and vagal reflexes) were compared using Fisher’s exact test. Because only two treatment groups and a limited number of predefined outcomes were compared, no formal correction for multiple comparisons was applied.

## 3. Results

**Figure 1:**
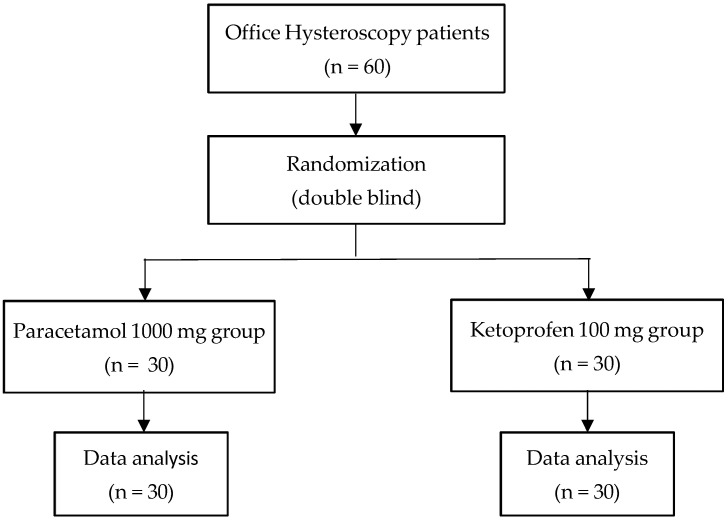
Research Flow Diagram.

A total of 60 female patients who underwent OH procedures at the RSUPN Dr. Cipto Mangunkusumo Kintani participated in this research. The research subjects were then divided into two groups, namely the group given the analgesic drug paracetamol 1000 mg orally and the group given ketoprofen 100 mg orally, as can be seen in the picture below (Figure 1).

Table 1 shows that most of the subjects were an average of 48.3 years old with an average procedure duration of 26 min. Most subjects were multiparous (55%), had not had menopause (53.3%), had no dysmenorrhea (80%), had no chronic pelvic pain (90%), and had no history of caesarean section (75%).

In Table 2, it can be seen that paracetamol and ketoprofen are not statistically significant for pain during the procedure (*p* value = 0.266), and cramping after the procedure 30 min (*p* value = 0.499).

As shown in Table 3, the subjects reported a high level of comfort during the OH procedure, with a median score of 9 (7–10). In addition, most subjects did not feel any side effects (95%) and all did not experience vagal reflexes (100%).

In Table 4, the VAS pain scores in the group given paracetamol 1000 mg and ketoprofen 100 mg did not have a significant difference in the confounding variables of procedure duration (*p* = 0.196), parity (*p* = 0.795), menopause (*p* = 0.121), dysmenorrhea. (*p* = 1.000), chronic pelvic pain (*p* = 0.671), and history of childbirth (*p* = 0.371).

## 4. Discussion

### 4.1. Subject Characteristics

There were 30 subjects who underwent OH procedures in each group. The majority of subjects did not have risk factors for increased OH pain, with a smaller ratio of subjects with nulliparity, menopause, dysmenorrhea, chronic pelvic pain, and having experienced a cesarean section. Procedure duration also tends to be short, with a median of 26 min. The median VAS pain score during the procedure was 2, and the cramping pain score after the procedure was 0, with a comfort level of 9, side effects of 5%, and no vagal reflex. The absence of risk factors for increased pain may explain the small pain scores and side effects that occurred. This can also be influenced by operator skill and other confounding factors.

The low pain score in this study was lower than the previous study by Zayed et al. [16], with the majority (46.5%) moderate (VAS 4–7) and low (33.9%) VAS 0–3 scores from 86 patients. They found that patients with nulliparity, cervical pathology, and patients with a procedure duration of more than 2 min were associated with intolerable pain (VAS 8–9). This contrasts with the majority of pain scores being low in this study, coupled with the relatively long duration of the procedure (26 min). A study by Guraslan et al. [19] of 303 women with OH also found moderate VASs in 38% of patients, with the determining factors being nulliparity, menopausal conditions, excessive flexion of the cervix and retroflexion of the uterus. However, in these studies, patients were not given premedication.

Another study by Coimbra et al. [20] found that good procedural tolerance was found in 85% of procedures, and poor tolerance in 15% of 1418 OH procedures. Tolerance rates in this study were better due to the administration of misoprostol before the procedure, even without administration of analgesics. This proves the importance of premedication with analgesics and cervical preparation before the procedure. The absence of significant risk factors for OH pain in this study could be caused by adequate premedication. Premedication masks the pain that would otherwise be present in patients with positive risk factors. Therefore, the results of risk factor analysis in this study can only be used to exclude confounding factors.

### 4.2. Paracetamol Compared with Ketoprofen as Analgesic for OH

Based on the unpaired T statistical test, it was found that there was no difference between pain scores during the procedure and the incidence of cramping 30 min after the procedure in patients given paracetamol 1000 mg compared with ketoprofen 100 mg orally. The median pain score during the procedure in the paracetamol group was 2 and the Ketoprofen group was 3, with a *p* value = 0.266. Meanwhile, the median cramping pain score 30 min after the procedure in both groups was 0.

Interestingly, our study included a higher proportion of postmenopausal women in the ketoprofen group (60.7%) compared with the paracetamol group (39.3%). Previous studies have shown that postmenopausal women tend to experience higher pain intensity during office hysteroscopy due to cervical stenosis and vaginal atrophy. Despite this imbalance, our findings demonstrated no significant difference in pain scores between the two groups. This suggests that ketoprofen may have provided effective pain suppression, even among postmenopausal women, in whom higher pain levels would otherwise be expected. The absence of increased pain in the ketoprofen group despite a higher proportion of postmenopausal subjects may indicate a potential benefit of ketoprofen in this subgroup. Regarding safety, the incidence and severity of adverse effects were mild and comparable between the two groups, suggesting that the analgesic efficacy of ketoprofen was achieved without a notable increase in side effects. Further studies with larger sample sizes and stratified subgroup analyses are needed to confirm these findings.

A total of 11% (*n* = 3) of subjects given ketoprofen experienced side effects. In the paracetamol group, no subjects experienced side effects. In the end, the subjective comfort scores in both groups were the same. Statistically, the two groups did not produce significantly different VASs, either overall, or taking into account risk factors such as procedure duration, parity, menopause, dysmenorrhea, chronic pelvic pain, and history of childbirth.

Administration of oral analgesics is recommended by RCOG 2023 [21] 60 min before the procedure, namely ibuprofen and/or paracetamol. However, there is no research that confirms its efficacy. A study reported that ketoprofen and paracetamol have onset times of 60–180 min and 45–180 min, respectively, placing the 60 min interval within the effective onset window for both drugs.

Previous trials comparing Paracetamol and ketoprofen in adults have demonstrated that both provide effective analgesia. Seymour et al. [22] reported similar pain reduction with 1000 mg paracetamol and 12.5–25 mg ketoprofen following third molar extraction, while Rosada-Kurasińska et al. [23] found no significant difference in postoperative pain or opioid consumption between 1000 mg paracetamol and 100 mg ketoprofen in vascular-surgery patients. Likewise, Karvonen et al. [24] observed comparable pain scores after hip replacement, although opioid use was slightly lower with ketoprofen. These findings suggest that both drugs provide similar analgesic efficacy across a range of pain intensities and surgical procedures.

Our results are consistent with these studies, showing no significant difference in pain scores between paracetamol and ketoprofen in the outpatient hysteroscopy setting, where this procedure is generally associated with mild to moderate discomfort. The equivalent efficacy observed in our study aligns with previous reports by Kokki et al. [25] and Hung et al. [26], which also found no meaningful difference between paracetamol and NSAID-based analgesia. Moreover, Issat et al. [17] compared intravenous misoprostol, ketoprofen, and placebo during hysteroscopy and reported significant pain reduction only with misoprostol, further suggesting that ketoprofen may not provide additional benefit in this context.

A review by Riemma et al. [27] also noted that while NSAIDs and paracetamol are routinely used during office hysteroscopy, a Cochrane analysis found insufficient evidence to support their effectiveness. The RCOG guidelines [21] state that the average VASs for pain during hysteroscopy is approximately 5.2, similar to menstrual pain at 5.5, underscoring that this procedure typically induces only moderate pain. Together, these findings support the interpretation that both oral paracetamol and oral ketoprofen provide comparable analgesia in low-to-moderate pain procedures such as office hysteroscopy, and that patient comfort may depend more on procedural and individual factors than on the choice between these two medications.

### 4.3. Side Effects of Paracetamol and Ketoprofen

Based on the Fischer’s exact test statistical test, there is no significant difference between the occurrence of side effects from using paracetamol 1000 mg and ketoprofen 100 mg orally. However, the number of samples with side effects was only 3 people, so the statistical test results could not be used. The majority of patients do not suffer side effects from the use of analgesics. There were only 3 patients (10%) who suffered side effects after using ketoprofen, in the form of symptoms of nausea and none suffered side effects after using paracetamol. This is in line with previous research conducted by De Saro et al. which found side effects of nausea and vomiting at a rate of around 13% [28]. Paracetamol is a very safe drug, and oral administration just once has a very small risk of side effects.

### 4.4. Strengths and Limitations of This Study

This study is the first to compare the efficacy of oral ketoprofen with oral paracetamol, specifically for pain management in OH procedures. The results of this study showed that the VAS values and cramping rates were quite low, so that overall, the procedure was considered quite comfortable for the patient. Moreover, the two drugs used in this study are very cheap and affordable, so their availability is very high.

This study has a limited sample size. Although the sample size is sufficient for comparative analysis of pain on paracetamol and ketoprofen, there are several other variables that cannot be analyzed. The absence of a no-premedication or placebo group, so the effectiveness of ketoprofen or paracetamol could not be assessed objectively. This study has samples from only one operator so it cannot be analyzed whether the efficacy and side effects of administering oral paracetamol 1000 mg or oral ketoprofen 100 mg in pain management during OH procedures are influenced by the operator. The exact 60 min interval between drug administration and the start of the procedure cannot be confirmed and may have varied slightly. However, medications were administered after registration, and the procedure began approximately 60 min later.

The surveillance for side effects was confined to during and immediately after the procedure. The study design did not include a longer-term follow-up to capture potential delayed adverse events. Future research should incorporate such a follow-up to provide a more comprehensive safety profile for these analgesics.

Non-normal data distribution for several outcome variables represents another limitation. The independent *t*-test was used. Given the equal group sizes and similar distributions, this was not expected to influence the study results. The subgroup analyses were exploratory and not adjusted for multiple comparisons. Nevertheless, all between-group comparisons were non-significant, and the overall interpretation of the findings remains unchanged.

## 5. Conclusions

There was no significant difference in VASs when administering 1000 mg paracetamol tablets orally compared with 100 mg ketoprofen tablets orally for pain management during the procedure and cramping 30 min after the OH procedure.

## Figures and Tables

**Table 1:** Characteristic of Subjects.

Variables	Paracetamol 1000 mg Tablet (*n* = 30)	Ketoprofen 100 mg Tablet (*n* = 30)	Total (*n* = 60)
Age (mean ± SD)	47.0 ± 13.20	49.6 ± 11.37	48.3 ± 12.28
Duration (minutes)	24.2 ± 8.44	27.8 ± 7.97	26.0 (10–45)
Parity (median [%])			
Nullipara	13 (48.1)	14 (51.9)	27 (100)
Multipara	17 (51.5)	16 (48.5)	33 (100)
Menopause (*n*)			
Yes	11 (39.3)	17 (60.7)	28 (100)
No	19 (59.4)	13 (40.6)	32 (100)
Dysmenorrhea (*n*)			
Yes	6 (50.0)	6 (50.0)	12 (100)
No	24 (50.0)	24 (50.0)	48 (100)
Chronic Pelvic Pain (*n*)			
Yes	2 (33.3)	4 (66.3)	6 (100)
No	28 (51.9)	26 (48.1)	54 (100)
History of caesarean section			
Yes	21 (46.7)	24 (53.3)	45 (100)
No	9 (60.0)	6 (40.0)	15 (100)

**Table 2:** Comparison of VASs for pain during the procedure and cramping 30 min.

	Drug Groups		*p* Value
	Paracetamol 1000 mg	Ketoprofen 100 mg	
Pain during procedure (VAS)	2 (0–8)	3 (0–6)	0.266
Cramping 30 min after procedure (VAS)	0 (0–5)	0 (0–4)	0.499

**Table 3:** VASs, side effects, comfort level, and vagal reflexes in both groups.

Variables	Paracetamol 1000 mg Tablet (*n* = 30)	Ketoprofen 100 mg Tablet (*n* = 30)	Total (*n* = 60)
Side Effect (*n*)			
Yes	0 (0.0)	3 (100.0)	3 (5.0)
No	30 (52.6)	27 (47.4)	57 (95.0)
Comfort Level (median)	9 (7–10)	9 (8–10)	9 (7–10)
Vagal Reflex (*n*)			
Yes	0 (0.0)	0 (0.0)	0 (0.0)
No	30 (50.0)	30 (50.0)	60 (100)

**Table 4:** The relationship of the confounding variables to the VAS pain scores in both groups.

	Paracetamol 1000 mg Tablet (*n* = 30)	Ketoprofen 100 mg Tablet (*n* = 30)	*p* Value
Duration of procedure (mean ± SD)			0.196
>26 min	2.38 ± 2.18	3.00 ± 1.91	
<26 min	2.35 ± 2.23	2.92 ± 2.11	
Parity (median)			
Nullipara and primipara	2 (0–6)	2 (0–6)	0.795
Multipara	3 (0–8)	4 (0–6)	
Menopause (median)			0.121
Yes	3 (0–8)	2 (0–6)	
No	2 (0–6)	4 (0–6)	
Dysmenorrhea (median)			1.000
Yes	2 (0–6)	4.5 (2–6)	
No	2 (0–8)	2.5 (0–6)	
Chronic Pelvic Pain (median)			0.671
Yes	2 (0–4)	3 (1–6)	
No	2 (0–8)	2 (0–6)	
History of caesarean section			0.371
Yes	3 (0–8)	4 (2–6)	
No	2 (0–6)	2.5 (0–6)	

## Data Availability

Data of this research is available if requested.

## Abbreviations

The following abbreviations are used in this manuscript:

NSAIDs	Nonsteroidal anti-inflammatory drugs
VAS	Visual analogue scale
TENS	Transcutaneous nerve stimulation
RCOG	Royal College of Obstetricians and Gynaecologists
OH	Office hysteroscopy
